# Association between Experiences of Different Types of Harassment or Derogatory Treatment and Sexual Harassment among Employees at a Large Swedish University

**DOI:** 10.3390/ijerph20010011

**Published:** 2022-12-20

**Authors:** Frida Pilgaard, Anette Agardh, Per-Olof Östergren, Gisela Priebe

**Affiliations:** Division of Social Medicine and Global Health, Department of Clinical Sciences Malmö, Clinical Research Centre, Lund University, 202 13 Malmö, Sweden

**Keywords:** sexual harassment, harassment, derogatory treatment, university employees, gender, academy, women’s health, workplace

## Abstract

Sexual harassment (SH) and other forms of mistreatment continue to be a significant problem at workplaces, leading to negative health and work-related outcomes. Previous studies have mainly examined SH and other types of workplace harassment separately. In this study we investigated whether harassment related to any of the seven Swedish legal grounds for discrimination (sex, transgender identity or expression, ethnicity, religion or other belief, disability, sexual orientation, or age) and derogatory treatment were associated with SH at a large Swedish university. Using cross-sectional survey data obtained from 33% of all staff, multivariable logistic regression analysis was performed to investigate associations between harassment, derogatory treatment, and SH. We found a sixfold increased risk of SH among women with experience of other forms of harassment and a three-times-higher risk among women with experience of derogatory treatment, indicating that SH co-occurs with other forms of mistreatment. This pattern was similar among men, although men reported lower prevalence of mistreatment. Our findings have implications for preventive strategies at academic workplaces indicating that issues related to the defence of power and various types of abusive behaviours, including SH, both need to be addressed to create more equal opportunities for all employees.

## 1. Introduction

Sexual harassment (SH) continues to be a significant problem at many workplaces, even though the general awareness of the problem increased after the #metoo movement in 2017 [1]. Other forms of mistreatment also exist at workplaces and share some features in common with SH [2]. As previous studies have mainly examined SH and other types of workplace harassment separately, research gaps exist concerning the relationship between various types of harassment, including SH [3]. In this study we investigated whether experiences of other types of *harassment*, defined as behaviours associated with one of the seven Swedish legal grounds for discrimination: sex, transgender identity or expression, ethnicity, religion or other belief, disability, sexual orientation, or age, and *derogatory treatment*, defined as insulting acts such as withholding information, derogatory comments, or exclusion, were associated with SH at a large Swedish university. The overarching term for all three types of undesirable behaviours (harassment, derogatory treatment, and SH) that we use in this paper is workplace mistreatment.

SH leads to multiple negative health- and work-related outcomes [3,4,5]. Individual and job-related consequences reported include anxiety, depression, decreased job satisfaction, absenteeism, and reduced productivity, not only affecting the individual but also causing considerable costs for organisations [6,7,8,9]. Negative consequences can be seen even in relatively mild cases of SH, such as sexist put-downs or offensive sexual remarks [10,11]. Typically, men perpetrate and women experience SH, although the reverse situation exists, as well as same-gender SH [12]. Reported levels of SH vary widely between studies and between countries due to factors such as differing definitions used, recall periods and representativeness of the research, as well as variation in cultural values and norms in different societies [4,13]. It has, for example, been observed that EU countries that rank high in terms of gender equality, such as Sweden, also tend to report higher prevalence rates of SH [14]. This could partly be due to greater awareness of SH in the society and of it being a fundamental rights abuse, leading to a greater willingness to report experiences of SH in a survey interview. A recent EU-wide survey on violence against women showed that every second woman (55%) in the EU has experienced sexual harassment at least once since the age of 15, among which 32% report that the perpetrator was someone from the workplace such as a colleague, a boss or a customer [14]. Not only are women more often subjected to SH than men, research findings suggest that they are also more negatively impacted by it [5,15,16]. For example, in one study, perceptions of workplace SH were associated with poor physical health among women, but not men [17].

Besides SH, other types of mistreatment such as discrimination, general harassment, bullying, incivility or microaggressions also exist at workplaces and impact negatively on health- and work-related outcomes [18,19,20,21,22,23,24]. As in the case of SH, the variety in definitions and terms used to capture these types of victimisation complicates comparisons across studies. In the European Working Conditions Survey (EWCS) from 2015, 12% of the participants reported verbal abuse and 6% reported humiliating behaviour in the month prior to the study [25]. Further, 5% reported bullying/harassment and 7% reported discrimination on the basis of gender, age, race, religion, nationality, disability, or sexual orientation during the 12 months prior to the study. The above-mentioned adverse social behaviours were experienced by women to a greater extent than by men [25].

There are different theories explaining why SH occurs [3,6]. A common understanding of SH is that it is one of the many expressions of gender-based violence [26]. This perspective regards sexual harassment as part of a continuum of violent behaviours and attitudes against women on the basis of gender, rooted in gender inequality, the abuse of power and harmful norms [26,27]. It has been suggested that the primary motive underlying all forms of harassment is the desire to protect or enhance social status when it seems threatened [28]. According to this view, SH would be motivated by a desire to protect or enhance a gender-based social status when it seems threatened, rather than by sexual desire. The defence of power in the form of identity or position has also been identified to be a shared commonality between SH and general harassment [2]. According to McDonald [3], “behaviours such as workplace bullying, mobbing, racial harassment and sex-based harassment, as well as SH, have hierarchical power relations at their core” (p. 12). Relevant in this context is the academic workplace setting, often filled with formal and informal power relations, which may influence the prevalence of SH and other types of mistreatment.

Besides the defence of power, McDonald points at other shared features of these workplace phenomena, such as the perception of the victim, the ambiguity of intent and the violation of organisational norms [3]. It has been reported that women rarely experience SH in isolation, but in combination with general non-sexualised mistreatment or incivility [29]. SH and gender discrimination are so closely linked that existing research often merges the two [17]. Further, discrimination or harassment based on social status might be difficult to disentangle, as identities and social status based on gender, class, race, age, disability, or sexuality are not always easily separable [17]. Clearly, there is an overlap between SH and various forms of other mistreatment at the workplace, yet few studies have examined these phenomena together [2]. Increased knowledge about how SH relates to other forms of workplace mistreatment would give valuable insights as to how SH can be understood in the context of multiple destructive workplace behaviours undermining equal opportunities for all employees [3]. This kind of insight can help improve preventive measures.

In the following section, we describe SH, harassment, and derogatory treatment according to Swedish legislation, which provides the definitions relevant to our study. In Sweden SH is mainly treated as a form of discrimination and falls under the Discrimination Act, defined as a conduct of a sexual nature that violates a person’s dignity [30]. SH can also constitute criminal acts, such as unlawful invasion of privacy or sexual molestation and is then regulated in the Swedish Penal Code. Other types of harassment, defined as situations when a person’s dignity is violated, must be related to one of the seven legal grounds for discrimination, i.e., “sex, transgender identity or expression, ethnicity, religion or other belief, disability, sexual orientation, or age” to fall under the Discrimination Act. Harassment or SH are behaviours that are unwelcome, and it is the victim of harassment who determines what is unwelcome or offensive. This means that there are no specific behaviours that define harassment or SH mentioned in the Discrimination Act, but rather it is the victim’s perception of a behaviour that is of importance. The perpetrator, however, must understand that the behaviour is unwelcome for it to be classified as harassment or SH according to the law [31].

Another form of mistreatment at the workplace regulated in Swedish law is defined as offensive or abusive actions towards one or more employees that may lead to ill health or to exclusion from the workplace community [32]. In this study we refer to this phenomenon as derogatory treatment, which includes experiences of insulting acts such as withholding information, derogatory comments, or exclusion. Swedish employers are obliged to pursue active prevention work against SH, harassment, and derogatory treatment at the workplace. However, undesirable behaviours continue to be a problem at Swedish workplaces and preventive measures need to be improved [33,34,35,36,37].

In summary, SH and various kinds of mistreatment exist at workplaces today with negative health- and job-related impacts on individuals and organisations. SH at the workplace seems to overlap with other forms of workplace mistreatment, and a better understanding of how these are related would be valuable when designing preventive measures. Workplace mistreatment is often rooted in hierarchical power relations. As the academic workplace is often characterised by formal and informal power relations, SH and other forms of mistreatment among the staff in this setting are of particular interest. Hence, the aim of this study was to examine whether experience of different forms of harassment and derogatory treatment was associated with SH among employees at a large public university in Sweden. Furthermore, a secondary aim was to investigate whether such associations differ by gender.

## 2. Methods

### 2.1. Data Collection

This study is part of the project “Tellus”, initiated at Lund University (LU), Sweden, 2018, with the aim of reinforcing preventive work against SH at the university [38]. As part of the Tellus project, a cross-sectional survey targeting both staff/PhD students and students was conducted at LU in 2019. The survey included questions related to SH, but also harassment related to the seven Swedish legal grounds for discrimination (sex, transgender identity or expression, ethnicity, religion or other belief, disability, sexual orientation or age) and derogatory treatment. Questions about the gender of the perpetrator and the relationship between the victim and the perpetrator were also included. The survey instrument used, as well as the overall results, such as prevalence of SH while employed at LU, are presented in detail elsewhere [39].

### 2.2. Study Population

In November 2019 a survey in both English and Swedish was sent by email to all staff at LU. PhD students are primarily employed by the university in Sweden and were therefore included as staff in our survey. The response rate was 33% (N = 2750). After exclusion of those with missing data on both sex and gender, those who did not answer any of the 10 questions on experiences of SH or those who did not specify when the SH took place, the final study population consisted of 2732 staff/PhD students.

A comparison between the participants and the target population by means of employment records found no major differences. However, a few observations were made; women were slightly over-represented among study participants and both men and women participants tended to be somewhat older compared to the target group. Further, a slight over-representation of staff with permanent employment (versus temporary) was seen [39].

### 2.3. Outcome Variable: Sexual Harassment and Sexual Violence

As the outcome variable, we used experiences of SH during the past 12 months at LU. The variable was based on the following list of ten behaviours, including one behaviour representing sexual violence, to measure experiences of sexual harassment or sexual violence: unwelcome suggestive looks or gestures; unwelcome soliciting or pressuring for ‘dates’; unwelcome ‘inadvertent’ brushing or touching; unwelcome bodily contact such as grabbing or fondling; unwelcome gifts; unwelcome comments; unwelcome contact by post or telephone; unwelcome contact online, for example, by social media or email; stalking; and attempts to conduct or the conduct of oral, vaginal, or anal sex or other equivalent sexual activity in which one did not participate voluntarily [39]. The study participants were asked if they had experienced any of these behaviours in connection with their employment at LU with the answer options: Yes, once; Yes, more than once; and No. All participants who answered yes to at least one of the above behaviours and stated that the behaviour/s occurred during the past 12 months were classified as exposed to SH during the past 12 months at LU.

### 2.4. Exposure Variables

#### 2.4.1. Harassment

The following text introduced the section about harassment in the survey: “The simplified definition of harassment provided in the Discrimination Act is that harassment occurs when someone is subjected to an act that violates their dignity and that this violation is associated with one of the seven grounds for discrimination: sex, transgender identity or expression, ethnicity, religion or other belief, disability, sexual orientation or age. Harassment can be both individual and isolated events as well as subtle, almost imperceptible events that continue over time, so-called microaggressions. It can also be a process that is ongoing and permeates the entire working life.” The participants were asked if they had experienced harassment as described above in connection with their work at LU during the past 12 months. The answer options were: No; Yes, once; Yes, more than once; and Yes, in the form of microaggressions or ongoing process. It was possible to select more than one answer to indicate whether the harassment was in the form of isolated acts, microaggressions or both. All participants answering Yes, once, Yes, more than once, or Yes, in the form of microaggressions or a process that is ongoing, were categorised as exposed to harassment and all others as not exposed. To obtain information about the type of harassment that was experienced, the participant was asked to select, from a list of the seven legal grounds for discrimination, the particular grounds to which the harassment could be attributed. It was possible to select several grounds.

The Swedish language uses the same word for both sex and gender. The official translation in the Discrimination Act is “sex”. Unless directly citing the Discrimination Act, we use “gender” in the subsequent sections of this paper.

#### 2.4.2. Derogatory Treatment

In the survey, the following information introduced the section with questions related to derogatory treatment: “This refers to derogatory or insulting acts directed at one or more employees. Examples of such acts include withholding information, derogatory comments and exclusion. The Swedish Work Environment Authority includes other examples such as the use of derogatory nicknames, shutting out, exclusion from meetings, unfair accusations, public personal attacks, and referring to someone in offensive terms in front of others. “The question was then put: Have you experienced derogatory treatment in conjunction with your work at Lund University during the past 12 months?” The answer options were: No; Yes, once; and Yes, more than once. All participants answering Yes, once or Yes, more than once were classified as exposed to derogatory treatment and all others as not exposed.

#### 2.4.3. Multiple Forms of Harassment or Derogatory Treatment

Each study participant could report experiences of several forms of harassment associated with the legal grounds for discrimination (sex, transgender identity or expression, ethnicity, religion or other belief, disability, sexual orientation, or age) as well as derogatory treatment. To explore experiences of multiple forms of harassment or derogatory treatment, we created a new variable summarising all forms reported by each individual, including experiences of derogatory treatment, and thereafter categorised all participants into three categories: Not exposed to any form of harassment or derogatory treatment; Exposed to *one* form of harassment or derogatory treatment; and Exposed to *two or more* forms of harassment or derogatory treatment. One participant had reported experiences of harassment once and two other participants had reported experiences of microaggressions but not linked to any specific form of harassment. These three participants were classified as exposed to one form of harassment or derogatory treatment.

#### 2.4.4. Sexual Harassment/Violence outside the University Setting

Survey participants were asked in two separate questions if they ever had been subjected to sexual harassment and/or sexual violence outside LU with the answer options: Yes, once; Yes, more than once; and No. All participants answering Yes, once or Yes, more than once to at least one of the two questions were categorised as exposed to SH/sexual violence outside LU and all others as not exposed.

### 2.5. Background Variables

Gender: Gender was based on two gender-related questions in the survey: ‘What gender were you assigned at birth?’ (female/male) and ‘what is your current gender identity?’ (Female/male/I do not identify as male or female). The answer to the second question was used to define participants as woman, man or non-binary; however, when the answer to this question was missing (N = 15), the answer to the first one was applied.

Age: Age was obtained by asking the participants what predefined age group they belonged to (≤30/31–40/41–49/50–59/≥60 years).

Form of employment: Participants were asked whether their form of employment was permanent or temporary.

Professional position: Professional position was specified according to nine types in the survey: “professor”, “senior lecturer”, “lecturer/teaching assistant”, “postdoc/associate senior lecturer”, “researcher/associate researcher”, “PhD student/research student”, “administrative staff/library staff”, “technical staff”, and “other”. For the purpose of analyses, some categories were grouped together. This resulted in the following six categories used in our analysis: “professors”, “senior lecturer”, “lecturer and researcher”, “PhD students”, “administrative and technical support staff”, and “others”.

Foreign background: Participants who were either born abroad or had two parents born abroad were categorised as individuals with a foreign background, in line with the definition used by Statistics Sweden [40]. Participants lacking information about parents’ country of birth were presumed to have Swedish background if they were born in Sweden.

### 2.6. Gender, Function and Formal or Informal Power Relation of Perpetrators

Participants who reported experiences of harassment and derogatory treatment, respectively, were asked about the gender of the perpetrator/perpetrators (male, female, non-binary or don’t know) as well as their function (a person employed at LU, a PhD student/research student at LU, a student at LU, or/and another person that I met through my work at LU) and the power relation between the perpetrator and the participant. If the perpetrator was a university employee, the following options were offered: (1) a person upon whom I was reliant; (2) a person in position of formal seniority to me; (3) a person in a position of power (formal or informal) over me; (4) a person over whom I am/was in a position of (formal or informal) power; and (5) another person at Lund university. The alternatives (1), (2), and (3) were categorised together as “dominant/upper position” in the analyses, indicating that the perpetrator had a dominant or higher power position in relation to the victim. If the perpetrator was a PhD student, the above-described alternatives (3), (4), and (5) were offered and categorised as representing a perpetrator’s “dominant/upper position”, “dependent/lower position” or “another person/relationship”, respectively, in relation to the victim in the analysis. As a participant could have been subjected to harassment and derogatory treatment by several perpetrators, it was possible to mark several options regarding gender, function and power relation. 

The same follow-up questions were asked about perpetrators of SH. However, as the questions about perpetrators referred to all experiences of SH, irrespective of time, we could not separate the information pertaining to perpetrators of SH during the last 12 months. However, the results regarding gender, function and power relation of perpetrators of SH (SH at any time) are published in a previous study presenting the overall results of the “Tellus” project [39].

The question regarding the gender of the perpetrator was not put to the participants who reported that they had been exposed to harassment in the form of microaggressions only (81 participants). Therefore, those 81 participants were excluded from the total number of participants exposed to harassment in the top section of Table 3 showing the gender of perpetrators.

### 2.7. Statistical Analysis

We used descriptive statistics to analyse the sociodemographic characteristics of the sample, prevalence of SH, harassment and derogatory treatment, as well as gender, professional position and power relation of the perpetrators of harassment and derogatory treatment. 

Association between background variables and SH was explored through a bivariate logistic regression analysis. A multivariable logistic regression analysis, including three different models adjusting for stepwise added background variables (age, foreign background and professional position) was performed to investigate associations between harassment, derogatory treatment or previous experience of SH outside LU and the outcome of SH. Interaction analyses were performed with dummy variables combining gender with exposure to harassment and gender with exposure to derogatory treatment, using men not exposed to harassment or derogatory treatment as the reference. When data allowed (not possible for all types of harassment due to small numbers), interaction analyses were performed by combining gender with specific types of harassment. The synergy indexes were calculated as proposed by Rothman, whereby a synergy index >1 indicates a synergistic effect, and a synergy index <1 an antagonistic effect [41].

We thought it important to present data from non-binary participants in the descriptive statistics, as this is rarely carried out due to often small numbers. By including them we make our data available for future pooled studies. However, due to small numbers, we excluded non-binary participants (24 out of 2732) from further analysis.

Statistical analyses were performed in Stata version 13.

### 2.8. Ethics

The data collection and use were approved by the Regional Ethical Review Board in Lund (Dnr 2018/350) and was in accordance with the 1964 Helsinki declaration and its later amendments or comparable ethical standards. 

## 3. Results

In total, 2732 individuals participated in the study, of which 57% were women, 42% men and 1% of non-binary gender. Generally, the men had higher professional positions than the women; 17% of the men versus 5% of the women reported that they were professors, and almost half of the women categorised themselves as administrative and technical staff compared to less than a third of the men. Regarding background factors, no other major differences were found between women and men. Table 1 shows the sociodemographic characteristics of the sample separately by gender.

### 3.1. Prevalence of SH, Harassment and Derogatory Treatment

Of all women, 8% reported having experienced SH at Lund University during the last 12 months compared to 3% of all men (Table 2). The three most common SH behaviours reported among both women and men were, in descending order, unwelcome comments, unwelcome suggestive looks or gestures, and unwelcome contact online; for example, by social media or email. Stalking was reported by six women and two men and attempted or completed rape by two women and no men.

Harassment related to any one of the seven Swedish legal grounds for discrimination (gender, transgender identity, sexual orientation, age, disability, religion and ethnicity) was reported by 10% and 4% of the women and men, respectively (Table 2). Harassment was more commonly reported as isolated events compared to microaggressions, except in the non-binary group (Table 2). The most common grounds for harassment in all groups were gender and age (Table 2). As many as 19% of the women and 9% of the men reported experience of derogatory treatment. Regarding single or multiple forms of harassment or derogatory treatment, 15% of all women reported experience of one form of harassment or derogatory treatment and 7% reported two or more forms. The equivalent numbers among men were 8% and 3%, respectively.

A vast majority of all women (61%) had previous experience of SH or sexual violence outside the university setting, not limiting the time window to the past 12 months. The corresponding number among men was 16% (Table 2).

The highest prevalence of experience of SH, harassment associated with the seven grounds for discrimination, and derogatory treatment was found in the non-binary gender group (Table 2).

### 3.2. Gender, Professional Function and Power Relation of the Perpetrator

The perpetrator of harassment or derogatory treatment was most often a man, irrespective of the gender of the exposed person. Of all participants exposed to harassment, 75% had been exposed by a man and 42% by a woman (it was possible to mark several options). Corresponding figures concerning derogatory treatment showed that 64% of the participants reporting experiences of derogatory treatment had been exposed by a man and 48% by a woman (see Table 3 and Table 4. Further, the perpetrator of harassment and derogatory treatment was most often another university employee with a dominant/higher position in relation to the victim. A total of 64% of the participants with experiences of harassment reported that the perpetrator was another university employee with a dominant/higher position in relation to them (Table 3). The corresponding figure for participants with experiences of derogatory treatment was 76% (Table 4).

### 3.3. Association between Background Variables and SH

Table 5 presents the associations between background variables, as well as exposure to other forms of harassment or derogatory treatment, and the outcome variable SH during the last 12 months at LU, separately by gender. Among women, a twofold risk of SH was found in the youngest age group (30 years and younger) compared to the oldest age group (60 years and above) (OR 2.4 95% CI 1.1–5.3). This pattern was not seen among men. No significant association was seen between professional position, form of employment or background (Swedish or foreign) and SH. Among both women and men, significant associations were found between experience of other forms of harassment or derogatory treatment and SH, as well as between experience of SH/sexual violence outside the university setting and SH at LU (Table 5).

### 3.4. Association between Harassment, Derogatory Treatment and SH

The multivariable logistic regression analysis showed a sixfold-increased risk of experiencing SH among women who had experienced harassment associated with any of the seven legal grounds for discrimination (Table 6). This elevated risk remained after adjusting for age, foreign background and professional position in Model 3 (OR 6.1 95% CI 3.9–9.4). The risk of experiencing SH among those who reported derogatory treatment was three times as high (OR 3.2 95% CI 2.1–4.7), and the risk of experiencing SH among those who had reported multiple forms of harassment or derogatory treatment (two or more forms of harassment associated with the seven legal grounds for discrimination or derogatory treatment) was eight times higher after adjusting for age, background (foreign or Swedish) and professional position (OR 7.5 95% CI 4.5–12.5). The pattern was similar among men, although with larger confidence intervals due to smaller numbers.

A clear association was also found between previous experience of SH/sexual violence outside the university setting (time not specified) and SH during the last 12 months at LU, among both women and men, with a doubled risk among women (OR 2.3 95% CI 1.4–3.6) and three times as high risk among men (OR 2.8 95% CI 1.3–5.9) after adjusting for age, background (foreign or Swedish) and professional position (Table 6).

### 3.5. Interaction Analysis

Interaction analyses performed with a dummy variable combining gender with exposure to harassment, and using men not exposed to harassment as the reference, indicated a moderate synergistic effect (SI 1.53) of female gender on the association between harassment and the outcome SH (Table 7). A similar analysis was performed by combining gender with exposure to derogatory treatment, showing no synergistic effect on SH (Table 7). Further interaction analyses were performed by combining gender with different types of harassment (only possible for age, gender and disability due to small numbers) regarding the effect on SH, but no modifying effects were found.

## 4. Discussion

This study investigated the association between experience of different types of harassment or derogatory treatment and SH among employees at a large Swedish university. We found a three-times-higher risk of SH among women with experience of derogatory treatment and a sixfold-increased risk of SH among women with experience of harassment, where such harassment was mainly attributed to gender and age. The highest risk of experiencing SH among women (eight times higher risk) was found among those who had reported multiple forms of harassment or derogatory treatment. The increased risks remained statistically significant after adjusting for age, foreign background and professional position. A similar pattern was seen among men, although men reported lower prevalence of mistreatment in general. Further, we found a moderate synergistic effect of female gender on the association between harassment and the outcome of SH, indicating that women with experiences of harassment had a higher risk than men with the same experience of also experiencing SH. The non-binary individuals were not included in the above-mentioned analysis because of low statistical power, but they reported the highest prevalence of SH, harassment and derogatory treatment.

Our results indicate that experiences of SH co-occur with other forms of mistreatment. This is in line with results from a study by Lim and Cortina finding an association and co-occurrence between sexual harassment and general incivility [29], as well as other research showing that individuals often report experiences of multiple forms of harassment and discriminatory behaviours at the workplace [21]. Our results also indicate that power relations play a role in situations of harassment and derogatory treatment, as most of the victims reported that the perpetrators were in a dominant/higher position in relation to themselves. Similarly, previous results from the same survey showed that perpetrators of SH were in a dominant/higher position in relation to the victim [39]. This result supports the theory that SH and other types of harassment may be driven by the need to defend or reinforce one’s power position [2,3]. We do not presuppose any causal direction between SH and other harassment or derogatory treatment. It is plausible that the defence of a social status or power position might involve a variety of behaviours directed towards the victim, thus generating experiences of multiple forms of mistreatment among the affected. The behaviours might vary as well as the perceptions of such behaviours, where victims’ perceptions might vary depending on previous experiences, social status or gender. For example, Berdahl and Aquino found a large gender difference regarding experiences of sexual behaviour at work; 46% of the men reported that they enjoyed it, compared to 10% of the women [42]. Other researchers have identified individual factors, such as age, gender, gender role, past experiences of sexual harassment and perceptions of management’s tolerance of sexual harassment, to be related to attitudes toward sexual harassment [43]. In this light, the identification of SH at the workplace can be an indicator of a larger problem, i.e., the existence of an unequal organisational culture where multiple types of abusive behaviours rooted in power imbalance and the defence of power contribute to unequal opportunities among employees. Our results have implications for designing preventive measures, suggesting that issues related to the defence of power and the various types of abusive behaviours including SH at a workplace both need to be addressed. On the other hand, it is possible that a positive spill-over effect could occur concerning other types of harassment behaviours if actions targeting SH behaviours are successful, given that the defence of power can be manifested in various ways by one individual.

In the university setting, there are various formal and informal hierarchical power relations that might trigger the defence of power irrespective of gender. However, underlying all other forms of power relations between staff is the informal and formal power imbalance between men and women. Men are generally attributed with higher status compared to the women in organisations, which gives men more informal and formal power [44]. Moreover, a formal power imbalance is evident in that men dominate the higher positions in the Swedish academic sector [45], which is also the case at Lund University, where, for example 72% of the professors were men in 2019 (personal communication human resources at LU) [45]. This power imbalance between genders is the context within which our study results should be understood. A majority of the perpetrators of harassment and derogatory treatment in our study were men, and data from the same survey shows that this also applies to perpetrators of SH [39]. The victims, on the other hand, were more often women; experiences of SH, harassment and derogatory treatment were about twice as common among women compared to men in our study. The formal and informal power imbalance between female and male university staff and its consequences should be systematically examined. Efforts to target and reduce workplace mistreatment at the academic workplace might mitigate the negative effects of gender inequality, although a true power balance is required to achieve real gender equality at academic workplaces. 

## 5. Strengths and Limitations of the Study

Our study has a large scope, including experiences of SH as well as other forms of harassment and derogatory treatment, which is a major strength. Studies examining SH together with other types of mistreatment are, to our knowledge, few, and thus the current study might contribute new valuable knowledge to the field of SH research. We base our analysis on a cross-sectional survey sent by email to all staff, which included PhD students and administrative staff at LU. The response rate was 33%, which might be considered low, but a comparison between the participants and the target population showed no major differences, and we believe our data represents the target population well. A limitation of questionnaire surveys concerning sensitive topics is that people with relevant experiences may choose not to participate because they do not want to be reminded of uncomfortable events. This might lead to an under-representation of those exposed. However, the opposite might also be true: people with no such experience might think that the survey is not relevant to them, causing an under-representation of those not exposed. We were able to control for the potential confounding effect of age, foreign background, and professional position in the analysis, which is an important strength in our study. This study is based on self-reported experiences of unwanted behaviours defined as SH, harassment, and derogatory treatment, which might be different from the number of cases meeting the legal definitions of SH and harassment. However, previous findings show that perceptions of SH and other types of workplace mistreatment are linked to ill health, and therefore self-reported experiences are relevant to our study [17,19]. In our study, we define SH using a list of ten unwanted behaviours. Generally, a definition of SH using a list of behaviours yields higher prevalence rates compared to using only one question [46]. Information about experiences of harassment and derogatory treatment, on the other hand, was obtained using one question, respectively. We introduced the questions related to harassment and derogatory treatment with explanatory texts, which for derogatory treatment also included examples of such behaviours. However, it is possible that if we had instead defined harassment and derogatory treatment using a list of behaviours it would have generated higher prevalence levels affecting the associations found between SH, harassment and derogatory treatment. A limitation of this study was that, due to low numbers, it was not meaningful to perform more detailed analysis of the non-binary gender group or specific harassment forms (sex, transgender identity or expression, ethnicity, religion or other belief, disability, sexual orientation or age). Further, as our study results are based on cross-sectional data, it is not possible to draw conclusions on the direction of causality.

## 6. Conclusions

In this study we found an increased risk of having experienced SH among those who also reported experiences of harassment or derogatory treatment, which indicates that experiences of SH co-occur with other forms of mistreatment. We also found that the prevalence of various types of mistreatment at the workplace is higher among women compared to men. Further, our results indicate that power plays a role in situations of harassment and derogatory treatment, supporting the theory that the defence of power may be a motivating factor underlying various types of harassment, including SH. Our findings might have implications for designing preventive strategies at academic workplaces, suggesting both the need to address issues related to the defence of power as well as measures to counteract various types of abusive behaviours, including SH. This knowledge can be useful when improving preventive measures to counteract workplace mistreatment, contributing to more equal opportunities for all at workplaces.

## Figures and Tables

**Table 1:** Sociodemographic characteristics of the sample, by gender.

		All (2732)		Women (1547)		Men (1161)		Non-Binary (24)	
		n	%	n	%	n	%	n	%
**Age groups**	≤30	335	12.3%	188	12.2%	144	12.4%	3	12.5%
	31–40	634	23.2%	373	24.1%	250	21.5%	11	45.8%
	41–49	770	28.2%	465	30.1%	300	25.8%	5	20.8%
	50–59	686	25.1%	364	23.5%	320	27.6%	2	8.3%
	≥60	307	11.2%	157	10.1%	247	12.7%	3	12.5%
	*Missing*	-	-	-	-	-	-	-	-
**Professional position**	Professors	285	10.4%	80	5.2%	203	17.5%	2	8.3%
	Senior lecturers	385	14.1%	189	12.2%	193	16.6%	3	12.5%
	Lecturers and researchers	457	17.4%	243	15.7%	227	19.6%	5	20.8%
	PhD Students	397	14.5%	222	14.3%	170	14.6%	5	20.8%
	Administrative and Technical staff	1098	40.2%	757	48.9%	334	28.8%	7	29.2%
	Other	90	3.3%	55	3.6%	33	2.8%	2	8.3%
	*Missing*	*2*	*0.1%*	*1*	*0.1%*	*1*	*0.1%*	*-*	*-*
**Employment form**	Permanent	1941	71.1%	1109	71.7%	817	70.4%	15	62.5%
	Temporary	741	27.1%	418	27.0%	314	27.0%	9	37.5%
	*Missing*	*50*	*1.8%*	*20*	*1.3%*	*30*	*2.6%*	*-*	*-*
**Background**	Swedish	2063	75.5%	1180	76.3%	868	74.8%	15	62.5%
	Foreign	667	24.4%	367	23.7%	291	25.1%	9	37.5%
	*Missing*	*2*	*0.1%*	*-*	*-*	*2*	*0.2%*	*-*	*-*

**Table 2:** Reported prevalence, by gender, of sexual harassment, harassment * and derogatory treatment.

		All (2732)		Women (1547)		Men (1161)		Non-Binary (24)	
		n	%	n	%	n	%	n	%
**Sexual harassment**		156	5.7%	119	7.7%	34	2.9%	3	12.5%
Harassment *	Harassment (any legal ground)	206	7.5%	155	10.0%	45	3.9%	6	25%
	Harassment as microaggressions only	81	3.0%	65	4.2%	11	1.0%	5	20.8%
	Harassment as isolated events only	107	3.9%	75	4.9%	31	2.7%	1	4.2%
	Harassment as microaggressions *and* isolated events	18	0.7%	15	1.0%	3	0.3%	-	-
	Harassment associated with **gender**	115	4.2%	97	6.3%	16	1.4%	2	8.3%
	Harassment associated with **transgender identity**	9	0.3%	1	0.1%	3	0.3%	5	20.8%
	Harassment associated with **sexual orientation**	18	0.7%	10	0.7%	5	0.4%	3	12.5%
	Harassment associated with **age**	58	2.1%	46	3.0%	12	1.0%	-	-
	Harassment associated with **disability**	14	0.5%	10	0.7%	4	0.3%	-	-
	Harassment associated with **religion**	10	0.4%	5	0.3%	4	0.3%	1	4.2%
	Harassment associated with **ethnicity**	38	1.4%	28	1.8%	10	0.7%	-	-
**Derogatory treatment**		403	14.8%	290	18.8%	108	9.3%	5	20.8%
Multiple forms of harassment or derogatory treatment **	Exposed to *one* form	334	12.2%	234	15.1%	96	8.3%	4	16.7%
	Exposed to *two or more* forms	141	5.2%	108	7.0%	29	2.5%	4	16.7%
**SH/sexual violence outside LU at any time**		1141	41.8%	944	61.0%	184	15.9%	13	54.2%

Note: * Harassment associated with the Swedish legal grounds for discrimination; ** summarising experiences of different types of harassment linked to the legal grounds for discrimination and derogatory treatment; the sexual harassment, harassment and derogatory treatment took place at Lund University during the last 12 months if not otherwise stated. SH, sexual harassment.

**Table 3:** Gender, function and power relation of the perpetrator/perpetrators * of harassment associated with the Swedish legal grounds for discrimination, by gender of exposed person.

Perpetrator Characteristics		Gender of Participants Exposed to Harassment **							
		Women (90)		Men (34)		Non-Binary (1)		All (125)	
		n	%	n	%	n	%	n	%
**Gender**									
	Male	70	77.8%	23	67.6%	1	100.0%	94	75.2%
	Female	34	37.8%	18	52.9%	-	-	52	41.6%
	Non-binary gender	1	1.1%	1	2.9%	-	-	2	1.6%
	Unknown gender	3	3.3%	1	2.9%	-	-	4	3.2%
		**Women (155)**		**Men (45)**		**Non-Binary (6)**		**All (206)**	
		**n**	**%**	**n**	**%**	**n**	**%**	**n**	**%**
**Function and power relation**									
**University employee**		134	86.5%	36	80.0%	6	100%	176	85.4%
	Dominant/higher position	104	67.1%	24	53.3%	3	50%	131	63.6%
	Dependent/lower position	9	5.8%	7	15.6%	1	16.7%	17	8.3%
	Other person/relationship	41	26.5%	15	33.3%	4	66.7%	60	29.1%
**PhD student/research student**		8	5.2%	10	22.2%	-	-	18	8.7%
	Dominant/higher position	2	1.3%	2	4.4%	-	-	4	1.9%
	Dependent/lower position	1	0.7%	3	6.7%	-	-	4	1.9%
	Other person/relationship	5	3.2%	7	15.6%	-	-	12	5.8%
**Student**		14	9.0%	6	13.3%	1	16.7%	21	10.2%
**Other person**		21	13.6%	5	11.1%	-	-	26	12.6%

Note: * Exposed persons could mark several options; ** information on gender of the perpetrator was missing for all participants reporting experiences of harassment in the form of microaggressions only (81 participants), and therefore they were excluded from the total number of exposed in the gender of perpetrator section. For more information, see Methods. The percentages are given as the percentages of ‘yes’ answers out of the total number of exposed persons in each gender group. The harassment took place during the last 12 months at Lund University.

**Table 4:** Gender, function and power relation of the perpetrator/perpetrators * of derogatory treatment by gender of exposed person.

Perpetrator Characteristics		Gender of Participants Exposed to Derogatory Treatment							
		Women (290)		Men (108)		Non-Binary (5)		All (403)	
		n	%	n	%	n	%	n	%
**Gender**									
	Male	186	64.1%	68	63.0%	3	60.0%	257	63.8%
	Female	142	49.0%	51	47.2%	1	20.0%	194	48.1%
	Non-binary gender	2	0.7%	2	1.9%	-	-	4	1.0%
	Unknown gender	3	1.0%	5	4.6%	-	-	8	2.0%
**Function and power relation**									
**University employee**		273	94.1%	98	90.7%	5	100.0%	376	93.3%
	Dominant/higher position	226	77.9%	76	70.4%	4	80.0%	306	75.9%
	Dependent/lower position	15	5.2%	10	9.3%	1	20.0%	26	6.5%
	Other person/relationship	56	19.3%	23	21.3%	2	40.0%	81	20.1%
**PhD student/research student**		9	3.1%	11	10.2%	-	-	20	5.0%
	Dominant/higher position	2	0.7%	2	1.9%	-	-	4	1.0%
	Dependent/lower position	3	1.0%	2	1.9%	-	-	5	1.2%
	Other person/relationship	5	1.7%	8	7.4%	-	-	13	3.0%
**Student**		15	5.2%	11	10.2%	-	-	26	6.5%
**Other person**		24	8.3%	7	6.5%	-	-	31	7.7%

Note: * Exposed persons could mark several options; the percentages are given as percentages of ‘yes’ answers out of the total number of exposed persons in each gender group. The derogatory treatment took place during the last 12 months at Lund University.

**Table 5:** Association between background variables, harassment *, derogatory treatment, experience of SH/sexual violence outside of Lund University and sexual harassment at Lund University, by gender. Bivariate logistic regression analysis.

Independent Variables (Missing)		Women n = 1547					Men n = 1161				
		n	n Exposed to SH	% Exposed to SH	OR	95% CI	n	n Exposed to SH	% Exposed to SH	OR	95% CI
**Age groups**	≤30	188	24	12.8%	2.4	1.1–5.3	144	4	2.8%	1.0	0.3–4.2
	31–40	373	34	9.1%	1.6	0.8–3.0	250	7	2.8%	1.0	0.3–3.6
	41–49	465	32	6.9%	1.4	0.7–2.6	300	12	4.0%	1.5	0.5–4.7
	50–59	364	20	5.5%	1.0	0.5–1.8	320	7	2.2%	0.8	0.2–2.8
	≥60	157	9	5.7%	1		147	4	2.7%	1	
**Professional position (women n = 1, men n = 1)**	Professors	80	10	12.5%	1		203	7	3.5%	1	
	Senior Lecturers	189	18	9.5%	0.7	0.3–1.7	193	6	3.1%	0.9	0.3–2.7
	Lecturers and researchers	243	17	7.0%	0.5	0.2–1.2	227	7	3.1%	0.9	0.3–2.6
	PhD Students	222	17	7.7%	0.6	0.3–1.3	170	3	1.8%	0.5	0.1–2.0
	Administrative and Technical staff	757	53	7.0%	0.5	0.3–1.1	334	10	3.0%	0.9	0.3–2.3
	Other	55	4	7.3%	0.6	0.2–1.9	33	1	3.0%	0.9	0.1–7.4
**Employment form (women n = 20, men n = 30)**	Permanent	1109	83	7.5%	1		817	22	2.7%	1	
	Temporary	418	34	8.1%	1.1	0.5–2.3	314	9	2.9%	1.1	0.8–1.6
**Background (men n = 2)**	Swedish	1180	86	7.3%	1		868	26	3.0%	1	
	Foreign	367	33	9.0%	1.3	0.8–1.9	291	8	2.8%	0.9	0.4–2.1
Harassment *	Not exposed	1392	77	5.5%	1		1116	26	2.3%	1	
	Exposed	155	42	27.1%	6.4	4.2–9.7	45	8	17.8%	9.1	3.9–21.4
**Derogatory treatment**	Not exposed	1257	71	5.7%	1		1053	20	1.9%	1	
	Exposed	290	48	16.6%	3.3	2.2–4.9	108	14	13.0%	7.7	3.8–15.7
Multiple forms of harassment **	Not exposed	1205	58	4.8%	1		1036	19	1.8%	1	
	Exposed to *one* form	234	30	12.8%	2.9	1.8–4.6	96	8	8.3%	4.9	2.1–11.4
	Exposed to *two or more* forms	108	31	28.7%	8.0	4.8–13.0	29	7	24.1%	17.0	6.5–44.7
**SH/sexual violence outside LU at any time**	Not exposed	603	26	4.3%	1		954	23	2.4%	1	
	Exposed	944	93	9.9%	2.4	1.55–3.8	173	11	6.0%	2.6	1.3–5.5

Note: * Harassment associated with the Swedish legal grounds for discrimination; ** summarising experiences of different types of harassment linked to the legal grounds for discrimination and derogatory treatment; CI, confidence interval; OR, odds ratio; SH, sexual harassment. All harassment and SH took place at LU during the last 12 months if not otherwise stated.

**Table 6:** Association between exposure to harassment *, derogatory treatment, experience of SH/sexual violence outside of Lund University and sexual harassment at Lund University, by gender. Odds ratios and 95% confidence intervals presented for three different multivariable logistic regression models. Women n = 1546, Men n = 1158.

Exposure Variables		Women n = 1546						Men n = 1158					
		Model 1 ^a^		Model 2 ^b^		Model 3 ^c^		Model 1 ^a^		Model 2 ^b^		Model 3 ^c^	
		OR	95% CI	OR	95% CI	OR	95% CI	OR	95% CI	OR	95% CI	OR	95% CI
Harassment *	Not exposed	1		1		1		1		1		1	
	Exposed	6.4	4.2–9.8	6.3	4.1–9.7	6.1	3.9–9.4	9.0	3.8–21.3	9.7	4.0–23.4	11.0	4.4–27.3
**Derogatory treatment**	Not exposed	1		1		1		1		1		1	
	Exposed	3.3	2.3–4.9	3.3	2.2–4.9	3.2	2.1–4.7	7.7	3.8–15.7	7.6	3.7–15.6	8.2	3.9–17.0
Multiple forms of harassment **	Not exposed	1		1		1		1		1		1	
	Exposed to *one* form	2.9	1.8–4.6	2.9	1.8–4.6	2.8	1.8–4.5	4.9	2.1–11.4	4.9	2.1–11.5	5.2	2.1–12.0
	Exposed to *two or more* forms	8.1	4.9–13.2	7.8	4.7–13.0	7.5	4.5–12.5	17.0	6.5–44.5	17.7	6.6–47.6	20.5	7.4–57.0
**SH/sexual violence outside LU at any time**	Not exposed	1		1		1		1		1		1	
	Exposed	2.4	1.6–3.8	2.3	1.5–3.6	2.3	1.4–3.6	2.6	1.3–5.5	2.6	1.3–5.5	2.8	1.3–5.9

Note: a: Model 1: crude; b: Model 2: adjusted for age and background (foreign or Swedish); c: Model 3: adjusted for age, background (foreign or Swedish) and professional position; * harassment associated with the Swedish legal grounds for discrimination; ** summarising experiences of different types of harassment linked to the legal grounds for discrimination and derogatory treatment; CI, confidence interval; OR, odds ratio; SH, sexual harassment. All harassment and SH took place at LU during the last 12 months if not otherwise stated.

**Table 7:** Interaction between gender and harassment *, and gender and derogatory treatment, regarding sexual harassment at Lund University.

	n	n Exposed to SH	% Exposed to SH	OR	95% CI	SI
Analysis (a) Gender and harassment						
**Man not exposed to harassment**	1116	26	2.3%	ref		
**Man exposed to harassment**	45	8	17.8%	9.06	3.8–21.4	
**Woman not exposed to harassment**	1392	77	5.5%	2.45	1.6–3.9	
**Woman exposed to harassment**	155	42	27.1%	15.58	9.2–26.4	
						1.53
Analysis (b) Gender and derogatory treatment						
**Man not exposed to derogatory treatment**	1053	20	1.9%			
**Man exposed to derogatory treatment**	108	14	13.0%	7.69	3.8–15.7	
**Woman not exposed to derogatory treatment**	1257	71	5.7%	3.09	1.9–5.1	
**Woman exposed to derogatory treatment**	290	48	16.6%	10.24	6.0–17.6	
						1.0

Note: * Harassment associated with the Swedish legal grounds for discrimination; SI, synergy index. All harassment and SH took place at LU during the last 12 months.

## Data Availability

Data cannot be shared publicly because of its sensitive nature. Data are available from Lund University (contact via the correspondent author) for researchers who meet the criteria for access to confidential data.
